# Detection and clinical manifestation of placental malaria in southern Ghana

**DOI:** 10.1186/1475-2875-5-119

**Published:** 2006-12-13

**Authors:** Frank P Mockenhaupt, George Bedu-Addo, Christiane von Gaertner, Renate Boyé, Katrin Fricke, Iris Hannibal, Filiz Karakaya, Marieke Schaller, Ulrike Ulmen, Patrick A Acquah, Ekkehart Dietz, Teunis A Eggelte, Ulrich Bienzle

**Affiliations:** 1 Institute of Tropical Medicine and International Health, Charité – University Medicine, Berlin, Germany; 2 Dept. of Medicine, Komfo Anoyke Teaching Hospital, School of Medical Sciences, Kwame Nkrumah University of Science and Technology, Kumasi, Ghana; 3 Presbyterian Mission Hospital, Agogo, Ghana; 4 Div. of International Health, Institute of Social Medicine, Epidemiology and Health Economy, Charité – University Medicine, Berlin, Germany; 5 Division of Infectious Diseases, Tropical Medicine and AIDS, Academic Medical Centre, Amsterdam, The Netherlands

## Abstract

**Background:**

*Plasmodium falciparum *can be detected by microscopy, histidine-rich-protein-2 (HRP2) capture test or PCR but the respective clinical relevance of the thereby diagnosed infections in pregnant women is not well established.

**Methods:**

In a cross-sectional, year-round study among 839 delivering women in Agogo, Ghana, P. *falciparum *was screened for in both, peripheral and placental blood samples, and associations with maternal anaemia, low birth weight (LBW) and preterm delivery (PD) were analysed.

**Results:**

In peripheral blood, P. *falciparum *was observed in 19%, 34%, and 53% by microscopy, HRP2 test, and PCR, respectively. For placental samples, these figures were 35%, 41%, and 59%. Irrespective of diagnostic tool, P. *falciparum *infection increased the risk of anaemia. Positive peripheral blood results of microscopy and PCR were not associated with LBW or PD. In contrast, the HRP2 test performed well in identifying women at increased risk of poor pregnancy outcome, particularly in case of a negative peripheral blood film. Adjusting for age, parity, and antenatal visits, placental HRP2 was the only marker of infection associated with LBW (adjusted odds ratio (aOR), 1.5 (95%CI, 1.0–2.2)) and, at borderline statistical significance, PD (aOR, 1.4 (1.0–2.1)) in addition to anaemia (aOR, 2.3 (1.7–3.2)). Likewise, HRP2 in peripheral blood of seemingly aparasitaemic women was associated with PD (aOR, 1.7 (1.0–2.7)) and anaemia (aOR, 2.1 (1.4–3.2)).

**Conclusion:**

Peripheral blood film microscopy not only underestimates placental malaria. In this highly endemic setting, it also fails to identify malaria as a cause of foetal impairment. Sub-microscopic infections detected by a HRP2 test in seemingly aparasitaemic women increase the risks of anaemia and PD. These findings indicate that the burden of malaria in pregnancy may be even larger than thought and accentuate the need for effective anti-malarial interventions in pregnancy.

## Background

Malaria in pregnancy continues to be a major public health problem in sub-Saharan Africa. Pregnant women are a specific risk group for *Plasmodium falciparum *infection, malaria and related consequences [1]. This increased risk is partially due to physiological alterations of immunity. Moreover, in pregnancy and by virtue of specific expression variants of the *P*. *falciparum *Erythrocyte Membrane Protein-1, parasite strains adhere to the syncytiotrophoblast, i.e. the surface lining the placental intervillous space. As immune recognition of and, thus, response to these parasites are low in primigravidae susceptibility and clinical manifestation are increased. With subsequent pregnancies, protective immune mechanisms gradually develop and expand, and manifestation of infection declines [2-4].

The placental sequestration of *P. falciparum *results in the accumulation of parasitized erythrocytes in the intervillous space, infiltration by inflammatory cells and release of pro-inflammatory mediators [5]. Clinical consequences of placental malaria, i.e. parasites and/or malaria pigment (haemozoin) present in placental blood, comprise maternal anaemia, low birth weight (LBW), preterm delivery (PD), and, consequently, increased perinatal and infant mortality [1,6-9]. In highly endemic regions, infected women are commonly asymptomatic [10]. Because of placental sequestration, peripheral blood film microscopy grossly underestimates the prevalence of placental malaria [11,12].

As with malaria in children, the prevalence and manifestation of malaria in pregnancy varies with transmission intensity, access to treatment, coverage and quality of antenatal services, and drug resistance, among others. The role of sub-microscopic *P. falciparum *infections, i.e. below the threshold of peripheral blood microscopy, is not well established [10,11,13-16]. The present study aimed at assessing prevalence and clinical consequences of *P. falciparum *infections detected in peripheral and placental blood by microscopy, histidine-rich-protein-2 (HRP2) capture test, and PCR among women delivering at a district hospital in rural southern Ghana. The performances of the diagnostic tests in a subset of 596 women have been reported previously [12]. Here, results from all 839 live-singleton deliveries are reported, focusing on the clinical manifestation of malaria in pregnancy.

## Methods

Agogo is a community of some 30,000 inhabitants located in the forested hills of Ashanti Akim North District. Subsistence farming, trade and mining are the main income sources in that region, and malaria is hyper- to holoenedemic [17]. In 1998, *P. falciparum *among women attending antenatal care at Agogo District Hospital was detected in 32% and 63% by microscopy and PCR assays of peripheral blood samples, respectively [10]. Then and at the time of study, chemoprophylaxis with pyrimethamine (PYR) was recommended in pregnancy; yet, *P. falciparum *mutations conferring PYR resistance are frequent in the area [18]. From January 2000 to January 2001, women attending Agogo Hospital for delivery were asked to participate in the present study and recruited after informed written consent was obtained. The study protocol was reviewed and approved by the Committee on Human Research Publications and Ethics, School of Medical Sciences, University of Science and Technology, Kumasi.

All women were clinically examined, socio-demographic data were documented, and venous peripheral blood collected into EDTA. Fever was defined as an axillary temperature >37.4°C. Following expulsion and after a small incision had been made into the maternal surface of the placenta, blood from the intervillous space was collected with a syringe containing EDTA. *P. falciparum *was screened for in both placental and peripheral blood samples by microscopy, HRP2 test, and PCR: malaria parasites were counted microscopically on Giemsa-stained thick blood films per 500 white blood cells for peripheral samples and per 100 high-power fields for placental samples in which the presence of leukocyte-associated haemozoin was also recorded. Based on placental thick blood film microscopy, the stage of placental infection was categorized as [5] early, only parasites visible; late, both parasites and pigment present; resolved, only pigment visible; and none, neither parasites nor haemozoin present. HRP2 was detected by rapid immuno-chromatographic tests (ICT Malaria P.f/P.v, Becton Dickinson, Germany). Following DNA extraction (QIAmp, Qiagen, Germany), nested *P. falciparum*-specific PCR assays were performed [19]. Plasma concentrations of chloroquine (CQ) and PYR were measured by ELISA assays [20] with limits of detection of 5 ng/mL and 10 ng/mL, respectively. Haemoglobin (Hb) was measured by a HemoCue photometer (Ångelholm, Sweden) and anaemia defined as Hb <11 g/dL. Crude birth weight and gestational age were assessed within 24 hours after delivery. LBW was defined as a birth weight <2500 g and PD as gestational age <37 weeks applying the Finnström score [21].

Geometric mean parasite densities (GMPDs) and 95% confidence intervals (95%CIs) were calculated. Continuous variables were compared between groups by t-test, analysis of variance, Mann-Whitney U test, and Kruskal-Wallis test as applicable. Associations between *P. falciparum *infection and anaemia, LBW, and PD were identified by χ^2 ^test, odds ratios (ORs) calculated, and adjusted for confounders by logistic regression models.

## Results

From January 2000 to January 2001, 889 delivering and consenting women were recruited and 922 babies were born (859 singles; 30 twin pairs, one set of triplets), of whom 22 (2.4%) were born dead or died within 24 hours after delivery. The characteristics of the 839 women with live-singleton deliveries are shown in Table 1. About half of the women originated from Agogo and about half had previously attended ≤3 antenatal care visits. Parity was strongly associated with LBW and PD, rates being more than double in primiparae (each, 26%) than in multiparae (Table 1). Fever was rare (3%). PYR in plasma was detected in 35% (median, 46 ng/mL; range, 11–1000) and CQ in 22% (median, 31 ng/mL; range, 6–1248).

**Table 1:** Characteristics of 839 women with live singleton deliveries

Parameter	All	Primiparae	Parae II & III	Multiparae (>III)	*P*
No. (%)	839 (100)	304/832 (36.5)	305/832 (36.7)	223/832 (26.8)	
Age (years; median, range; *n *= 827)	25 (15–47)	21 (15–36)	25 (18–40)*	33 (22–47)*	< 0.0001
Residence in Agogo (%, No.)	48.9 (410)	48.7 (148/304)	54.8 (167/305)	41.3 (92/223)	0.009
Proportion without school degree (%, No.)	15.4 (128/829)	8.6 (26/302)	14.6 (44/302)*	26.0 (57/219)*	< 0.0001
Axillary temperature (°C; mean ± SD; *n *= 826)	36.4 ± 0.5	36.5 ± 0.6	36.4 ± 0.5	36.4 ± 0.5	0.32
Fever (%, No.)	2.8 (23/826)	4.0 (12/300)	1.7 (5/302)	2.7 (6/129)	0.22
Haemoglobin (g/dL; median; range)	11.5 (4.6–16.8)	11.4 (4.6–16.4)	11.6 (5.8–16.8)	11.5 (4.7–15.6)	0.28
Anaemia (haemoglobin <11 g/dL)	35.2 (295)	38.5 (117/304)	32.5 (99/305)	35.0 (78/223)	0.30
No. of previous ANC visits (median, range, *n *= 821)	4 (0–12)	3 (0–11)	4 (0–12)*	4 (0–10)	0.01
Proportion with ≤3 ANC visits (%, No.)	47.7 (392/821)	52.5 (156/297)	43.1 (129/299)*	47.9 (105/219)	0.07
Caesarean section (%, No.)	22.1 (185)	21.7 (66/304)	19.0 (58/305)	26.5 (59/223)	0.12
Birth weight (g; median, range; *n *= 838)	2950 (1280–4500)	2785 (1280–4000)	3000 (1600–4250)*	3100 (1500–4500)*	<0.0001
Gestational age (weeks; median, range; *n *= 831)	38.4 (20.0–43.1)	38.4 (20.0–43.1)	39.0 (32.0–43.1)*	39.4 (27.0–42.1)*	0.0001
Low birth weight (LBW)	16.0 (134/837)	26.0 (79/304)	10.9 (33/304)*	9.4 (21/223)*	< 0.0001
Preterm delivery	18.8 (156/831)	26.3 (80/304)	16.0 (48/300)*	12.2 (27/221)*	< 0.0001
Proportion with chloroquine in plasma (%, No.)	22.4 (182/821)	27.9 (83/298)	19.1 (57/299)*	19.4 (42/217)*	0.02
Proportion with pyrimethamine in plasma (%, No.)	35.0 (287/821)	35.6 (106/298)	33.8 (101/299)	35.9 (78/217)	0.85

*, difference to primiparae, *P *< 0.05; ANC, antenatal care

### Detection and prevalence of Plasmodium falciparum

In all 839 peripheral samples, *P. falciparum *was detected in 19.0%, 34.1%, and 52.9% by microscopy, HRP2 test, and PCR, respectively. For placental samples, these figures were 34.9%, 40.8%, and 59.4%; placental haemozoin was seen in 30.9%. As reported before [12], sensitivity of peripheral blood film microscopy in detecting placental infection was poor. It was 50% (95%CI, 44–59) and 32% (56–63) taking the results of placental microscopy and PCR assays, respectively, as reference (specificities, 98% (96–99) and 100% (99–100)). For HRP2 tests on peripheral blood samples, these sensitivities were 78% (73–82; specificity, 89% (86–92)) and 56% (51–60; specificity, 98% (95–99)). PCR assays on peripheral samples achieved a sensitivity of 97% (94–99; specificity, 71% (69–75)) for microscopically confirmed placental parasitaemia and of 87% (83–89; specificity, 96% (93–98)) with PCR-proven placental infection as reference. Irrespective of diagnostic technique, *P. falciparum *declined in prevalence with parity as did placental parasite density (Table 2). Also, the presence of haemozoin decreased with parity and, consequently, late infections predominated in primiparae and early infections in multiparae (Table 2). Non-falciparum species were rare (six *Plasmodium malariae*, five *Plasmodium ovale*) and not related to parity.

**Table 2:** Prevalence of *Plasmodium falciparum *in 832 delivering women according to parity

Parameter	Prevalence of *Plasmodium falciparum *(%)			*P*
		
	Primiparae	Parae II & III	Multiparae	
No.	304	305	223	
***Peripheral blood***				
Diagnosic mean				
Microscopy	27.0	16.4*	11.7*	< 0.0001
HRP2 test	45.4	30.5*	23.8*	< 0.0001
PCR	59.2	52.1	46.2*	0.01
GMPD (μL, 95%CI)	740 (481–1137)	343 (189–623)*	593 (263–1336)	0.12
***Placental blood***				
Diagnostic mean				
Microscopy	46.1	33.1*	22.0*	< 0.0001
Haemozoin	42.4	29.8*	16.6*	< 0.0001
HRP2 test	50.7	37.4*	32.3*	< 0.0001
PCR	65.1	59.7	52.0*	0.01
GMPD (/HPF; 95%CI)	1.2 (0.8–1.8)	0.6 (0.4–1.1)	0.4 (0.2–0.7)*	0.01
Stage of infection				
None	47.4	57.7*	70.9*	
Resolved	6.6	9.2	7.2	
Late	35.9	20.7*	9.4*	
Early	10.2	12.5	12.6	< 0.0001

HRP2, histidine-rich-protein 2; GMPD, geometric mean parasite density; 95%CI, 95% confidence interval; HPF, high power field; *, difference to primiparae, *P *< 0.05

### Factors influencing the presence of placental malaria

Placental haemozoin was the clinically most relevant marker of infection and factors influencing its presence were analysed. In multivariate analysis, primiparity, low age, and delivery in the rainy season were independently associated with haemozoin (Table 3). In women with >3 antenatal care visits, haemozoin was somewhat less common (28.2%, 121/429) than in women with fewer visits (33.7%, 132/392; *P *= 0.09). PYR in plasma was associated with a slight reduction in the prevalence of haemozoin only (27.9%, 80/287 *vs*. 32.4%, 173/534; *P *= 0.18). In primiparae, this difference was pronounced and statistically significant (31.1%, 33/106 *vs*. 47.9%, 92/192; *P *= 0.005).

**Table 3:** Risk factors of placental haemozoin

Factor	Haemozoin detected (%, fraction)	Univariate analysis		Multivariate analysis	
			
		OR (95%CI)	*P*	OR (95%CI)	*P*
Parity					
>1	24.2 (128/528)	1		1	
1	42.4 (129/304)	2.3 (1.7–3.2)	< 0.0001	1.5 (1.0–2.2)	0.04
Age-group (years)					
>30	17.4 (33/190)	1		1	
26–30	27.0 (57/211)	1.8 (1.1–2.9)	0.02	1.7 (1.0–2.8)	0.04
21–25	33.9 (82/242)	2.4 (1.5–4.0)	0.0001	2.1 (1.3–3.5)	0.003
≤20	45.7 (84/184)	4.0 (2.4–6.6)	<0.0001	3.0 (1.7–5.2)	0.0002
Season					
Dry	27.1 (109/402)	1		1	
Rainy*	34.3 (150/437)	1.4 (1.0–1.9)	0.02	1.4 (1.1–2.0)	0.02

OR, odds ratio: 95%CI, 95% confidence interval; *, rainy season, May-October

### Association between diagnosis of malaria and its clinical manifestation

Irrespective of diagnostic tool, *P. falciparum *infection increased the risk of maternal anaemia (Table 4). A positive peripheral blood film or a positive PCR result were neither associated with LBW nor with PD. In peripheral blood samples, the HRP2 test best discriminated women with and without malaria-associated adverse foetal outcome (Table 4). Importantly, in women with a negative peripheral blood film, a positive HRP2 test indicated risk increases of 71%, 79%, and 114% for LBW, PD, and anaemia, respectively. A positive PCR signal in this sub-group was associated only with anaemia. Further stratification revealed that in case of a positive peripheral result exclusively by PCR (*n *= 160), the proportions of LBW (15%) and PD (14%) were similar to the figures in *de facto *non-infected women (*n *= 382; 14%, *P *= 0.7; and 17%, *P *= 0.4). Still, the prevalence of anaemia was increased (39% *vs*. 25%, *P *= 0.002).

**Table 4:** Prevalences of low birth weight, preterm delivery, and maternal anaemia according to diagnosis of *Plasmodium falciparum*

Diagnostic tool	No.	Low birth weight			Preterm delivery			Anaemia		
		
		%	OR (95%CI)	*P*	%	OR (95%CI)	*P*	%	OR (95%CI)	*P*
***Peripheral blood***										
Microscopy										
Negative	680	15.8			18.4			32.9		
Positive	159	17.0	1.1 (0.7–1.8)	*ns*	20.3	1.1 (0.7–1.8)	*ns*	44.7	1.6 (1.1–2.4)	0.005
HRP2 test										
Negative	553	14.3			16.5			29.5		
Positive	286	19.4	1.4 (1.0–2.1)	0.06	23.1	1.5 (1.1–2.2)	0.02	46.2	2.1 (1.5–2.8)	<0.0001
PCR										
Negative	395	14.2			17.9			25.6		
Positive	444	17.6	1.3 (0.9–1.9)	*ns*	19.6	1.1 (0.8–1.6)	*ns*	43.7	2.3 (1.7–3.1)	<0.0001
HRP2 test if microscopy negative										
Negative	542	14.2			16.5			29.3		
Positive	138	21.9	1.7 (1.0–2.8)	0.03	26.1	1.8 (1.1–2.9)	0.01	47.1	2.1 (1.4–3.2)	<0.0001
PCR if microscopy negative										
Negative	394	14.2			17.9			25.6		
Positive	286	17.9	1.3 (0.9–2.0)	*ns*	19.1	1.1 (0.7–1.6)	*ns*	43.0	2.2 (1.6–3.1)	<0.0001
***Placental blood***										
Microscopy										
Negative	546	14.3			17.7			28.8		
Positive	293	19.2	1.4 (1.0–2.1)	0.07	20.8	1.2 (0.8–1.8)	*ns*	47.1	2.2 (1.6–3.0)	<0.0001
Haemozoin										
Negative	580	13.6			16.5			27.8		
Positive	259	21.3	1.7 (1.2–2.6)	0.005	23.9	1.6 (1.1–2.3)	0.01	51.7	2.8 (2.0–3.8)	<0.0001
HRP2 test										
Negative	497	12.9			16.0			27.0		
Positive	342	20.5	1.8 (1.2–2.6)	0.003	22.8	1.6 (1.1–2.2)	0.01	47.1	2.4 (1.8–3.3)	<0.0001
PCR										
Negative	341	13.5			17.5			23.8		
Positive	498	17.7	1.4 (0.9–2.1)	*ns*	19.7	1.2 (0.8–1.7)	*ns*	43.0	2.4 (1.8–3.3)	<0.0001
Stage of infection										
None	482	13.5			16.7			27.0		
Resolved	64	20.3	1.6 (0.8–3.3)	*ns*	25.0	1.7 (0.9–3.2)	*ns*	42.2	2.0 (1.1–3.5)	0.01
Late	195	21.6	1.8 (1.1–2.8)	0.009	23.6	1.5 (1.0–2.4)	0.04	54.9	3.3 (2.3–4.7)	<0.0001
Early	98	14.3	1.1 (0.6–2.1)	*ns*	15.5	0.9 (0.5–1.7)	*ns*	31.6	1.3 (0.8–2.1)	*ns*

OR, odds ratio, 95%CI, 95% confidence interval; *ns*, not significant, *P *> 0.1

Peripheral blood parasite densities (GMPD, 558/μL; 95%CI, 404–772) correlated negatively with Hb (log_10 _parasite density, r = -0.18, *P *= 0.02) and gestational week (r = -0.18, *P *= 0.03) but not with birth weight (r = -0.06; *P *= 0.5). Placental parasite density (GMPD, 0.78/high power field; 95%CI, 0.06–1.03) did not show such correlations.

On the basis of placental sampling, again, microscopic and PCR detection of parasitaemia were not associated with LBW or PD whereas a positive HRP2 result was. Nevertheless, haemozoin was the marker of infection most strongly associated with all clinical consequences (Table 4). Likewise, late infections were found to be the most deleterious ones while patients with early infections clinically were similar to non-infected women. Haemozoin was also associated with the largest reduction in birth weight (median, 2850 g (range, 1280–4160) *vs*. 3000 g (1420–4500), *P *< 0.0001) which was smaller in case of a positive HRP2 test (2880 g (1420–4160) *vs*. 3000 (1280–4500), *P *= 0.0009) or a positive PCR result (2900 (1280–4350) *vs*. 3000 g (1500–4500), *P *= 0.04), and smallest if placental parasites were seen by microscopy (2900 g (1280–4160) *vs*. 2970 g (1420–4500), *P *= 0.02). Likewise, any diagnosis of placental *P. falciparum *was associated with significantly reduced Hb levels, but the reduction was largest in the presence of haemozoin (median, 10.8 g/dL (range, 4.6–15.6) *vs*. 11.7 g/dL (4.7–16.8), *P *< 0.0001). The week of gestation was not associated with infection, irrespective of diagnostic mean applied (data not shown).

Adjusting the above associations between *P. falciparum *infection and clinical manifestation for the confounders age, parity, and number of antenatal care visits did not substantially change the findings, however, statistical significance in terms of LBW and PD was reduced (Table 5).

**Table 5:** Adjusted risk estimates for low birth weight, preterm delivery, and maternal anaemia according to diagnosis of *Plasmodium falciparum*

Positive diagnosis	Low birth weight		Preterm delivery		Anaemia	
			
	aOR (95%CI)	*P*	aOR (95%CI)	*P*	aOR (95%CI)	*P*
***Peripheral blood***						
Microscopy	0.9 (0.6–1.5)	*ns*	1.1 (0.7–1.7)	*ns*	1.6 (1.1–2.3)	0.01
HRP2 test	1.2 (0.8–1.8)	*ns*	1.4 (0.9–2.0)	*ns*	2.0 (1.5–2.7)	<0.0001
PCR	1.1 (0.8–1.7)	*ns*	1.1 (0.7–1.5)	*ns*	2.1 (1.6–2.9)	<0.0001
HRP2 test if microscopy negative	1.5 (0.9–2.5)	*ns*	1.7 (1.0–2.7)	0.03	2.1 (1.4–3.2)	0.0002
PCR if microscopy negative	1.2 (0.8–1.9)	*ns*	1.0 (0.7–1.6)	*ns*	2.0 (1.4–2.8)	<0.0001
***Placental blood***						
Microscopy	1.1 (0.7–1.7)	*ns*	1.1 (0.7–1.6)	*ns*	2.2 (1.6–2.9)	<0.0001
Haemozoin	1.3 (0.8–1.9)	*ns*	1.3 (0.9–1.9)	*ns*	2.8 (2.0–3.9)	<0.0001
HRP2 test	1.5 (1.0–2.2)	0.04	1.4 (1.0–2.1)	0.055	2.3 (1.7–3.2)	<0.0001
PCR	1.2 (0.8–1.9)	*ns*	1.1 (0.8–1.6)	*ns*	2.4 (1.7–3.3)	<0.0001
Stage of infection						
Resolved	1.5 (0.8–3.0)	*ns*	1.5 (0.8–2.9)	*ns*	2.0 (1.1–3.4)	0.02
Late	1.2 (0.8–1.9)	*ns*	1.2 (0.8–1.9)	*ns*	3.4 (2.3–4.9)	<0.0001
Early	1.1 (0.6–2.1)	*ns*	1.0 (0.5–1.8)	*ns*	1.2 (0.8–2.0)	*ns*

aOR, adjusted odds ratio, 95%CI, 95% confidence interval; *ns*, not significant, *P *> 0.1. Odds ratios are adjusted for age (years), parity (log_10_), and antenatal care (≤, > 3 visits).

## Discussion

Placental malaria is an important cause of both LBW and PD, which, in turn, are major determinants of neonatal and infant mortality [22-24]. Annually, up to 200,000 infant deaths are attributed to malaria in pregnancy [25]. However, malaria in pregnancy frequently lacks overt clinical signs and its diagnosis is complicated by placental parasite sequestration.

In the present study, the vast majority of *P. falciparum*-infected delivering women were asymptomatic, and microscopy of thick films from peripheral blood missed more current infections than it detected. Roughly half of the women with microscopically proven placental parasitaemia had a negative peripheral blood film. The observed superior sensitivities of peripheral blood PCR or HRP2 assays are essentially in accord with previous reports [11,14,26,27] and underscore that a negative peripheral blood film in pregnant women in endemic areas is hardly informative. In addition, the present study shows that in contrast to peripheral blood microscopy and PCR, HRP2 detection indicates an increased risk of poor pregnancy outcome and that sub-microscopic infections in pregnancy are associated with maternal and foetal morbidity.

Placental histology is considered the "gold standard" of malaria diagnosis in pregnancy for epidemiological or study purposes, however, due to limited technical expertise it is rarely available in endemic areas. Examination of thick films of blood samples obtained by placental incision is comparatively easier. As compared to histology, sensitivity and specificity of this method have been reported to be 76% and 99%, respectively, and thus to be in the range of microscopy of blood films produced by scraping the wall of the incision or by spinning down erythrocyte pellets from biopsy washings [28]. The detection of haemozoin on placental thick blood films likely is influenced by this difference in sensitivity and also neglects pigment deposition in placental structures. Consequently, staging placental infection based on placental thick blood films findings [5] – although deduced from the histological classification after Bulmer *et al*. [29] – is not fully comparable to histology and needs further evaluation. Nevertheless, haemozoin may be considered a "black box" in recording episodes of malaria in pregnancy even though the time span during which pigment can be found after resolved infection is unknown. In Thailand, placental haemozoin was observed among women with preceding malaria during the first trimester [30] but in Malawi, haemozoin concentrations were not elevated in women who had infections early in pregnancy [31]. For clinical use, haemozoin detection is practically unhelpful as it can be performed only at delivery, however, for epidemiological and intervention studies, it appears feasible and cost-effective.

The sensitivities of the HRP2 test (78%) or PCR (97%) in detecting microscopically proven placental parasitaemia in peripheral blood were substantially higher than the one of peripheral blood film microscopy (50%). Improving performances of the peripheral blood tests with increasing placental parasite density have been described previously [12]. Because *P. falciparum *secretes HRP2, this protein can be detected in peripheral blood despite placental sequestration. In the present study, the specificities of the HRP2 test and of PCR were rather low when placental parasitaemia was taken as the reference. However, this probably is due to undetected parasites: when a positive placental PCR result was set as reference, specificities of HRP2 test and PCR on peripheral blood samples approached 100%. Similarily, the few studies comparing histology to PCR indicate a specificity of the latter of 100% [32,33]. Nevertheless, it should be kept in mind that PCR assays can remain positive for some days after curative treatment (and HRP2 tests for some weeks) [34,35].

Poor pregnancy outcome, e.g. LBW or PD, in this cross-sectional study at delivery was neither associated with a positive peripheral blood result of microscopy nor of PCR. This may be explained by the low sensitivity of the former, and the very high one of the latter. The HRP2 test with in-between sensitivity appears to be a practical compromise between sensitivity on the one hand and detecting clinically relevant infection on the other. Importantly, in seemingly aparasitaemic women, i.e. in those with a negative peripheral blood film, a positive HRP2 test was associated with anaemia, LBW and PD. Infections detected beyond that exclusively by PCR had no discernible effect on pregnancy outcome but were still associated with reduced maternal Hb. Previous studies on that topic produced conflicting results: in Ghana but not in Sudan, Mozambique, and Cameroon, sub-microscopic peripheral blood infections as detected by PCR were associated with low Hb levels or anaemia [10,13,15,16]. Similar to the findings of the present study, *P. falciparum *as identified by peripheral blood microscopy or PCR in Burkina Faso was not associated with LBW or birth weight whereas the latter was reduced in case of a positive peripheral blood HRP2 dipstick [14]. Women found to be *P. falciparum *positive by PCR exclusively exhibited no increased risk of LBW in Malawi [11]. One study from Kenya reported that malaria as assessed by peripheral blood film was stronger associated with intrauterine growth retardation than were peripheral PCR or detection of circulating parasite antigen [36]. Differing results in these studies may be due to sample sizes, endemicity, and techniques applied. Taken together, one conclusion could be that PCR assays also detect low-level infections which produce no major foetal impairment. However, this interpretation needs caution as the course of sub-microscopic *P. falciparum *infection in pregnancy, e.g. abortive, chronic or progressive, is unknown. Considering the common fluctuations of parasite density [37], the temporal pattern of the manifestation of malaria in pregnancy [7] and the results presented here any infection detected should be considered potentially harmful (and treated). Even sub-microscopic infections detected exclusively by PCR were associated with maternal anaemia which may impair physical performance and increase the risk of PD [38].

The detection of haemozoin and placental HRP2 to similar extents were univariately associated with anaemia, LBW and PD but the reductions of Hb and birth weight were most pronounced in women with placental pigment. The association between placental haemozoin and LBW is well documented, e.g. in Senegal [39]. In contrast, in Malawi, haemozoin concentrations were neither associated with PD nor with intrauterine growth retardation [31], and in Tanzania, the role of placental pigment in LBW and PD waned when considering mononuclear placental infiltration and high peripheral parasite density, respectively [8]. The latter authors argued that haemozoin rather reflects the severity of a recent infection than having direct detrimental effects on foetal growth. However, haemozoin concentrations have been reported to correlate with the placental expression of tumour necrosis factor [40], and recent investigations indicate that pigment is not inert but exhibits direct clinical effects [41].

After adjusting for age, parity, and antenatal visits, the risk of maternal anaemia remained to be increased in the presence of *P. falciparum*, irrespective of diagnostic tool. However, in terms of placental diagnosis, only HRP2 was associated with LBW and PD, the latter at borderline statistical significance. Also, seemingly aparasitaemic women with HRP2 in peripheral blood exhibited an increased risk of PD. Infection detected by microscopy or PCR were not correlated with birth outcome. This unexpected but not uncommon finding [6,7,42] may reflect the importance of the temporal pattern of infection [7] and the greater contribution of various other factors to adverse foetal outcome. For instance, HIV infection, malnutrition, genetic traits, smoking, and a low socio-economic status are risk factors for poor pregnancy outcome [43-45] but these factors were either not assessed or were rare. Recent data indicate a median HIV prevalence of 2.7% among antenatal care attendees in Ghana [46]. Still, malaria may effect poor pregnancy outcome indirectly *via *maternal anaemia [38,47].

The effects of antenatal care and of PYR in plasma were modest in that infection was not consistently prevented by either factor. Reasons for the limited impact of PYR chemoprophylaxis likely include poor compliance (only 35% of the women had detectable PYR) and drug resistance. Already in 1998, 81% and 36% of *P. falciparum *isolated from pregnant women in Agogo exhibited a *dihydrofolate reductase *resistance core mutation and the high resistance triple mutation, respectively [18]. From 2005 on, intermittent preventive treatment (IPT) with sulfadoxine-pyrimethamine is operative at Agogo Hospital, and results from a survey in pregnancy in 2006 are being awaited. Optimistically, with extended antenatal services and coverage, and IPT, a reduction in the burden of malaria in pregnancy in this area should be achievable.

## Conclusion

In conclusion, placental malaria commonly is missed by standard microscopy of peripheral blood films. Whereas positive results of PCR seem not to be related to poor pregnancy outcome, detection of HRP2 is capable of identifying *P. falciparum *infections associated with maternal and foetal morbidity. This is particularly true for women with a negative peripheral blood film. These findings are of importance for the implementation and evaluation of preventive measures for pregnant women in endemic areas and likely also for the assessment of response to treatment. The incorporation of sensitive rapid test devices such as HRP2 tests into antenatal services would be desirable. So far, the costs of IPT with SP are lower than the price of a dipstick of €1 ore more. However, cost-benefit considerations may change once SP has to be replaced because of intensifying drug resistance by more expensive drugs. In an operational sense, a dipstick in the second and third trimester may identify women in whom antimalarial interventions failed and who are at increased risk of maternal and foetal morbidity.

## Authors' contributions

FPM, GBA, and UB designed the study. CvG, RB, KF, IH, FK, MS, UU, FPM and PAA were responsible for patient recruitment, and clinical and parasitological examinations. ED and FPM did the statistical analyses; TAE measured drug concentrations. FPM wrote the paper with major contributions of the other authors.
